# Discovery of Novel Recurrent Mutations and Clinically Meaningful Subgroups in Nodal Marginal Zone Lymphoma

**DOI:** 10.3390/cancers12061669

**Published:** 2020-06-23

**Authors:** Jiwon Koh, Insoon Jang, Seongmin Choi, Sehui Kim, Ingeon Jang, Hyun Kyung Ahn, Cheol Lee, Jin Ho Paik, Chul Woo Kim, Megan S. Lim, Kwangsoo Kim, Yoon Kyung Jeon

**Affiliations:** 1 Department of Pathology, Seoul National University Hospital, Seoul 03080, Korea; jiwonsophia@gmail.com (J.K.); sulparangi@naver.com (S.K.); fejhh@hanmail.net (C.L.); cwkim@snu.ac.kr (C.W.K.); 2 Department of Pathology, Seoul National University College of Medicine, Seoul 03080, Korea; ingun0116@naver.com; 3 Division of Clinical Bioinformatics, Biomedical Research Institute, Seoul National University Hospital, Seoul 03080, Korea; isjang1324@gmail.com (I.J.); sm.alex.choi@gmail.com (S.C.); 4 Seoul National University College of Medicine, Seoul 03080, Korea; 5 Cancer Research Institute, Seoul National University, Seoul 03080, Korea; ahk0917@snu.ac.kr; 6 Department of Pathology, Seoul National University Bundang Hospital, Seongnam-si 46371, Korea; paikjhmd@gmail.com; 7 Department of Pathology and Laboratory Medicine, University of Pennsylvania Perelman School of Medicine, Philadelphia, PA 19104, USA; Megan.Lim@pennmedicine.upenn.edu

**Keywords:** malignant lymphoma, marginal zone lymphoma, genetics, whole exome sequencing, RNA sequencing

## Abstract

Nodal marginal zone lymphoma (NMZL) is a rare B-cell neoplasm, the genetic and transcriptomic landscape of which are unclear. Using high-throughput sequencing for whole-exome and transcriptome, we investigated the genetic characteristics of NMZL in a discovery cohort (*n* = 8) and validated their features in an extended cohort (*n* = 30). Novel mutations in *NFKBIE* and *ITPR2* were found in 7.9% (3/38) and 13.9% (5/36), respectively, suggesting roles for the NF-κB pathway and B-cell-receptor-mediated calcium signaling pathway in the pathogenesis of NMZL. RNA-seq showed that NMZLs were characterized by an aberrant marginal zone differentiation, associated with an altered IRF4-NOTCH2 axis and the enrichment of various oncogenic pathways. Based on gene expression profile, two subgroups were identified. Compared with subgroup 1, subgroup 2 showed the following: the significant enrichment of cell cycle-associated and MYC-signaling pathways, a more diverse repertoire of upstream regulators, and higher Ki-67 proliferation indices. We designated two subgroups according to Ki-67 labeling, and subgroup 2 was significantly associated with a shorter progression-free survival (*p* = 0.014), a greater proportion of large cells (*p* = 0.009), and higher MYC expression (*p* = 0.026). We suggest that NMZL has unique features and, in this study, we provide information as to the heterogeneity of this enigmatic entity.

## 1. Introduction

Nodal marginal zone lymphoma (NMZL) is a rare mature B-cell neoplasm that accounts for about 1–2% of all B-cell non-Hodgkin lymphoma (NHL) [1,2]. Compared with its related entities—splenic marginal zone lymphoma (SMZL) and extranodal marginal zone lymphoma (EMZL)—NMZL is characterized by the primary involvement of nodal sites [3]. Most patients present with localized or generalized lymphadenopathy, and bone marrow (BM) involvement is noted in 28–62% of patients [4]. NMZL mimics the nodal involvement of EMZL and SMZL, but the lack of disease-defining markers makes it difficult to diagnose this entity [4,5]. Among the three types of marginal zone lymphomas (MZLs), NMZL is the least known in terms of genetic alterations and pathogenesis. To date, only two studies have evaluated the genetic characteristics of NMZL by high-throughput sequencing. Spina et al. found frequent genetic alterations that affect chromatin modifiers and the NOTCH pathway and suggested that *PTPRD* mutation is the key genetic feature of NMZLs [6]. In another study performed on Swiss patients with NMZL, Pillonel et al. reported recurrent *BRAF* mutations, suggesting that *BRAF* mutations are diagnostically and therapeutically useful [7]. Despite the partially overlapping genetic features of NMZLs reported by the two studies (altered chromatin remodeling and the NOTCH pathway), the key findings of each study—*PTPRD* and *BRAF* mutations—were not reproducible. This suggests the pathogenic heterogeneity of NMZL, emphasizing the need for investigation of its genetic alterations. In addition, most previous molecular studies on NMZLs involved European or western population [8,9,10], thus the molecular genetic characteristics of NMZL in other ethnicities are unknown.

By a comprehensive review of the clinical and histopathological features, we established a cohort of patients with NMZL and performed the first genetic study of NMZLs in Asians. We performed whole-exome sequencing (WES) and transcriptomic analysis using various gene expression and knowledge-based bioinformatics platforms and validated the findings in an extended cohort. We identified novel genetic alterations, dysregulated pathways, and distinct subgroups of NMZLs.

## 2. Results

### 2.1. Clinicopathological Characteristics of the NMZL Cohorts

A total of 38 NMZLs were included in this study (Table 1). Detailed information on the clinicopathological features and histopathological characteristics of the cohort are shown in Table S1 and Figure S1.

### 2.2. Mutational Landscape of NMZL and Identification of Candidate Genes

**Figure 1:**
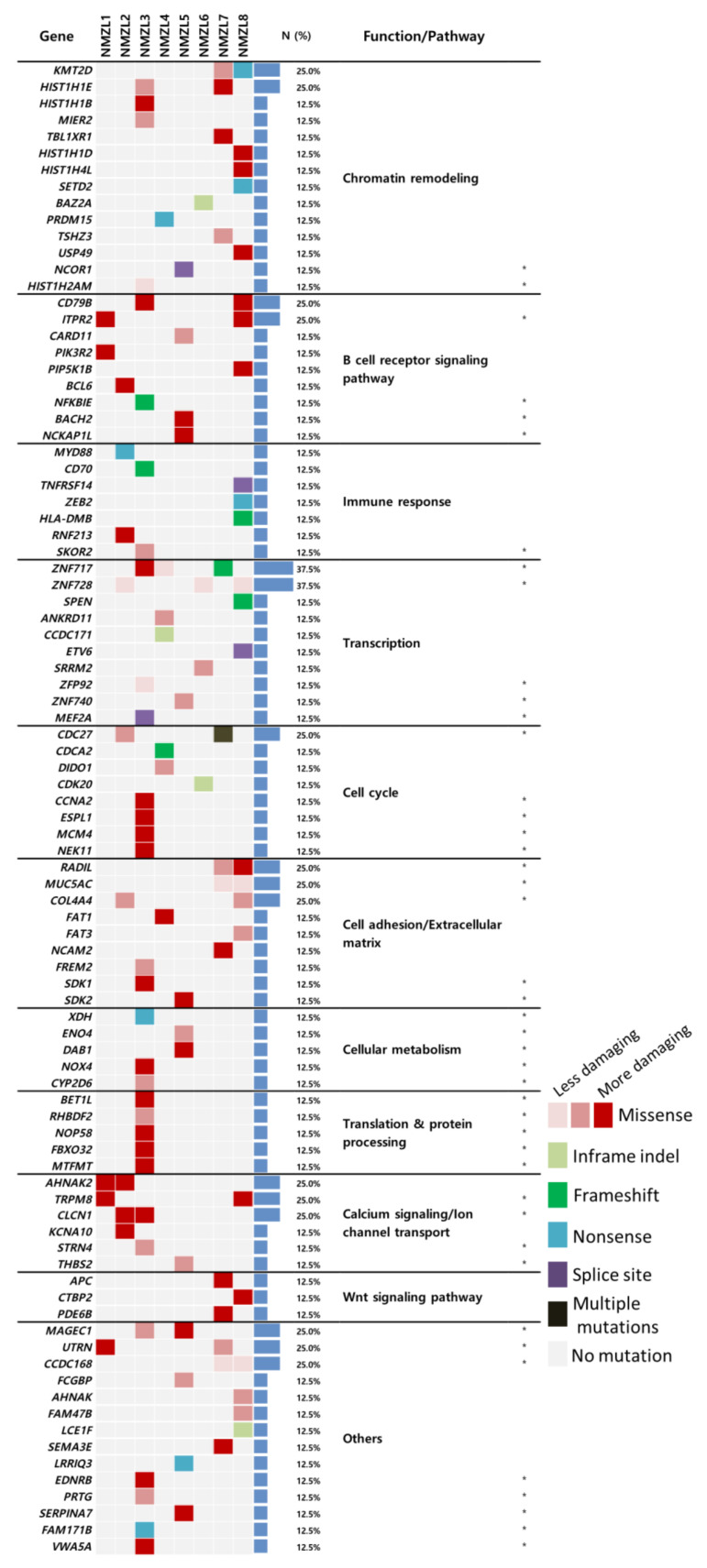
Mutational landscape of nodal marginal zone lymphoma (NMZL). A total of 92 candidate NMZL genes (CNGs) were grouped according to their functional relevance. Consistent with previous reports on the genetics of NMZL, genes related to chromatin remodeling and the B-cell receptor (BCR)-signaling pathway were the most frequently mutated. Other genes, presumably related to the immune response, transcription, and cell cycle were also found to be altered in NMZLs (asterisk: novel genes with mutations not previously found in NMZLs).

A total of 390 non-synonymous variants in 367 genes and a mean number of variants per case of 48.8 (range 13–96) were found (Table S2). We selected 92 candidate NMZL genes (CNGs) based on the following criteria: (i) genes with mutations in at least two of the eight patients, (ii) genes with mutations in NMZL3 or NMZL5 (which had matched germline controls), or (iii) genes with mutations in at least one case and implicated in B-cell lymphomas (Figure 1). Of 92 CNGs, mutations in genes involved in chromatin remodeling (*KMT2D*, 25.0%; *HIST1H1E*, 25.0%; *HIST1H1B*, 12.5%) and B-cell receptor (BCR)-signaling and related pathways (*CD79B*, 25.0%; *CARD11*, 12.5%; *BCL6*, 12.5%) were recurrently affected.

**Figure 2:**
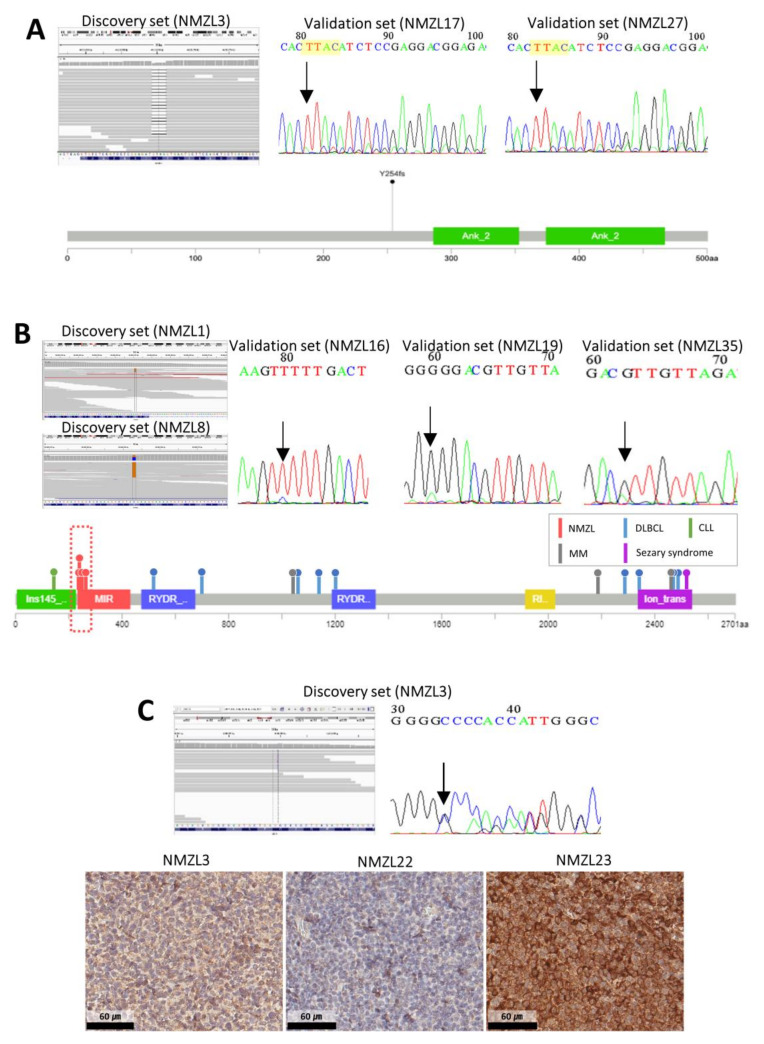
Validation of novel mutations found in the current study. (**A**) A 4-bp deletion frameshift mutation of *NFKBIE* (Y254fs) was found in NMZL3 by whole-exome sequencing (WES) (left), and direct sequencing identified another two patients in the validation set (NMZL17 and NMZL27) with the identical mutation (right). In total, three out of 38 (7.9%) patients with NMZL harbored this mutation (bottom). (**B**) WES revealed two patients in the discovery set with *ITPR2* mutations (left). Direct sequencing was performed to identify alterations in the other NMZL patients (right). Of the NMZLs, 13.9% (5/36) had mutations in *ITPR2*, all of which were predicted to be damaging and to affect the MIR domain of *ITPR2* (red box), whereas all *ITPR2* mutations in other types of lymphomas affected different sites (bottom). (**C**) NMZL3 patient harbored a frameshift mutation in CD70 (left), which was confirmed by direct sequencing (right). Immunohistochemistry (IHC) images with loss or attenuation of CD70 expression (NMZL3 and NMZL22) and intact CD70 expression (NMZL23) (bottom) are shown (abbreviations: multiple myeloma (MM)).

**Figure 3:**
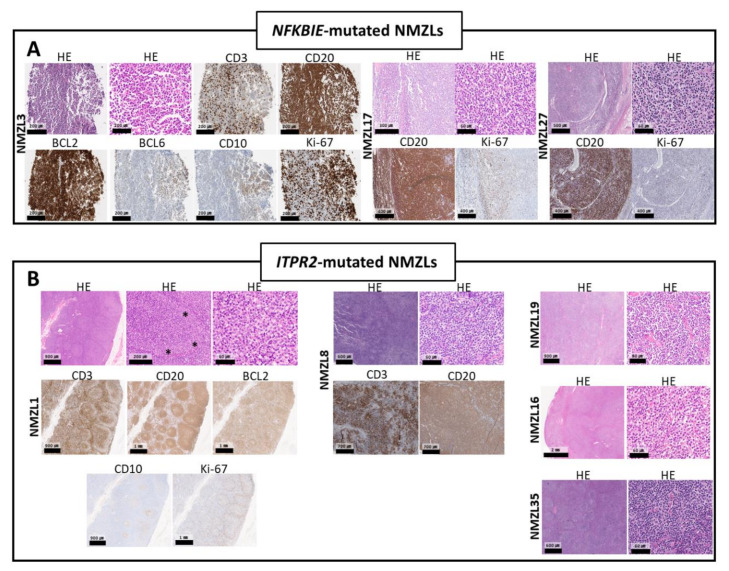
Histologic features of NMZLs harboring *NFKBIE* or *ITPR2* mutations. (A) NMZLs with *NFKBIE* mutations had varying proportions of medium-to-large B-lymphocytes intermingled with small cells. Heterogeneous medium-sized, CD20-positive, plasmacytoid B-cell proliferation, intermingled with small lymphocytes and a few large cells, was observed in a neck lymph node (LN) of NMZL3 (left), which also exhibited high Ki-67 positivity. The LNs of NMZL17 (middle) and NMZL27 (right) were composed of occasional large blastic cells intermingled with small lymphocytes. (B) All cases with *ITPR2* mutations showed varying degrees of marginal zone differentiation with proliferation of monocytoid B-cells, sometimes with clear cytoplasm. NMZL1 (left) exhibited prominent marginal zone hyperplasia (asterisk) (abbreviations: hematoxylin and eosin (H&E)).

We also identified 46 novel genes with mutations not reported previously in NMZLs. *NFKBIE* Y254fs mutation was detected in one patient (NMZL3); this is a loss-of-function mutation, resulting in deregulation/activation of NF-κB signaling [11]. Two other NMZL samples (NMZL17 and NMZL27) with the same *NFKBIE* mutation were found in the validation set by direct sequencing; thus, in total, *NFKBIE* Y254fs was observed in 7.9% (3/38) of the NMZLs (Figure 2A). The clinical and histopathologic features of the *NFKBIE*-mutated NMZLs are shown in Table S3 and Figure 3A. Briefly, patients with *NFKBIE*-mutated NMZLs tended to have a higher International Prognostic Index (IPI) score, more advanced disease and a higher Ki-67 proliferation index.

Mutations in *ITPR2* have not been reported previously in NMZLs. In this study, two patients in the discovery set had point mutations in *ITPR2* (NMZL1, V239A; NMZL8, R265P). Three more patients in the validation set harbored *ITPR2* point mutations; thus, 13.9% (5/36) of NMZLs had *ITPR2* mutations (Figure 2B). Notably, all five *ITPR2* mutations found in NMZLs were clustered within the MIR domain of *ITPR2*, whereas *ITPR2* mutations previously reported in other types of lymphomas at lower frequencies were located at other sites (Figure 2B). In silico prediction using five tools indicated that all *ITPR2* mutations observed in NMZLs would result in functional alterations (Table S4). The histopathologic features of *ITPR2*-mutated NMZLs are presented in Figure 3B. All cases had varying degrees of marginal zone (MZ) differentiation with the proliferation of small-to-medium sized B-lymphocytes, whereas NMZL8 and NMZL16 showed some intermingled large blastic cells. The clinical features of patients with *ITPR2*-mutated NMZLs were not significantly different from those with wild-type *ITPR2* (Table S5).

Several genes related to the immune response (*MYD88*, 12.5%; *CD70*, 12.5%; *TNFRSF14*, 12.5%) were also mutated, suggesting linkage between immune dysregulation and the pathogenesis of NMZL. *CD70* A80fs mutation, a novel variant not in the COSMIC database or the literature, was found in NMZL3 and validated by direct sequencing (Figure 2C). No additional case with a *CD70* mutation was found in the validation cohort; however, 61.1% (22/36) of NMZL cases showed loss or attenuated expression of CD70 by immunohistochemistry (IHC) (Figure 2C), indicating alteration of the immune-stimulatory molecule CD70 in NMZLs.

In addition, we manually searched for common gene fusions reported in B-cell NHLs, however, no fusions involving these genes were found.

### 2.3. Transcriptomic Features of NMZLs in Comparison with Non-Neoplastic LNs

**Figure 4:**
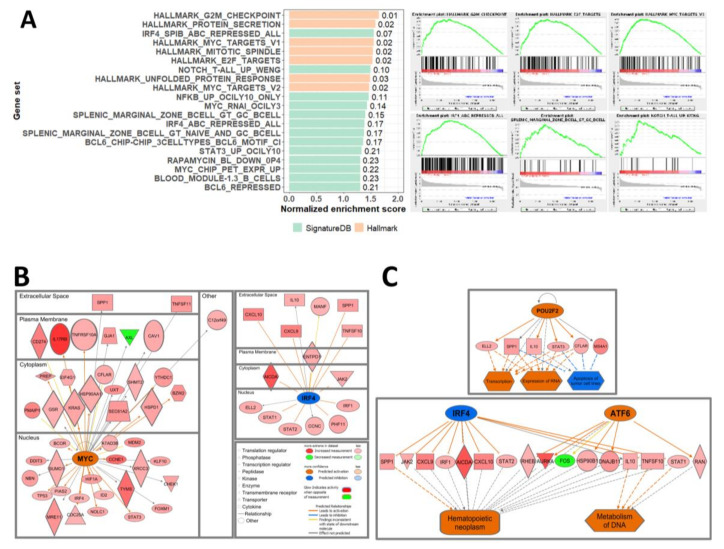
Gene set enrichment analysis (GSEA) and network analysis of NMZL samples compared to non-neoplastic LNs. (**A**) Significantly enriched gene sets in NMZLs and their normalized enrichment score (NES) are plotted. False discovery rate (FDR) *q*-values are shown to the right side of the bars (left). Representative enrichment plots of key gene sets are shown (right). (**B**) Mechanistic networks for the key upstream regulators MYC and IRF4 are shown. (**C**) IRF4 and ATF6 were predicted to cooperate, resulting in altered DNA metabolism and development of hematopoietic neoplasm, according to regulator effect analysis, and the B-cell-specific TF POU2F2 was implicated in transcription and inhibition of apoptosis.

Considering the small number of sample replicates used in this study, the gene set permutation gene set enrichment analysis (GSEA) algorithm [12] was used to identify the pathways enriched in NMZLs compared with non-neoplastic lymph nodes (LNs). Selected gene sets from MSigDB (http://software.broadinstitute.org/gsea/msigdb) and the SignatureDB collection (https://lymphochip.nih.gov/signaturedb/) [13] were used for the analyses. In total, we found 20 significantly enriched gene sets in NMZLs (Figure 4A). Gene sets representing cell cycle/proliferation and oncogenic pathways associated with MYC, NOTCH, NF-κB, and STAT3 were notably enriched in NMZLs. Sets of genes reportedly upregulated in splenic MZ B-cells compared with naïve B-cells or germinal center (GC) B-cells (SPLENIC_MARGINAL_ZONE_BCELL_GT_GC_BCELL and SPLENIC_MARGINAL_ZONE_BCELL_GT_NAIVE_AND_GC_BCELL) [14] were significantly enriched (normalized enrichment score (NES) = 1.40 and 1.37; false discovery rate (FDR) *q*-value = 0.15 and 0.17). Notably, sets of genes upregulated after *IRF4* and/or *SPIB* knockdown in activated B-cell (ABC)-type diffuse large B-cell lymphoma (DLBCL) cell line [15] (IRF4_ABC_REPRESSED_ALL and IRF4_SPIB_ABC_REPRESSED_ALL; NES = 1.38 and 1.55; FDR *q*-value = 0.17 and 0.07) were also significantly enriched in NMZLs. These findings suggest that NMZLs can be characterized by their accelerated cell cycle and proliferation, possibly driven by a number of oncogenes and transcription factors (TFs).

### 2.4. Major Upstream Regulators and Their Roles in the Pathogenesis of NMZL

According to the log_2_FC ratios, 2696 differentially expressed genes (DEGs) were identified: 78 were downregulated and 2618 upregulated compared with LNs. We performed upstream regulator analysis on DEGs using Ingenuity Pathway Analysis (IPA^®^) to identify upstream regulators that can account for the gene expression profile (GEP) of NMZLs. Among the significant upstream regulators (Table S6), MYC was notably activated regulator (*z* = 3.291, *p* = 0.008), and IRF4 was inhibited (*z* = −2.913, *p* = 0.004); IRF4 was predicted to affect AICDA, IRF1, JAK2, STAT1 and STAT2 (Figure 4B). MITF had the highest *z*-score (*z* = 4.796, *p* = 3.47E-04); other molecules related to the cell cycle and apoptosis, including FOXM1, E2F6, TP53, NUPR1, and the histone modifier KDM5B, were identified (Figure S2). Subsequent regulator effect analysis predicted the biological effects of upstream regulators (Table S7). The B-cell specific TF POU2F2 was predicted to inhibit tumor cell apoptosis and enhance transcription, and IRF4 in combination with ATF6 was predicted to play an important role in the development of hematopoietic neoplasm (Figure 4C).

### 2.5. Characterization of NMZL Subgroups according to GEPs

**Figure 5:**
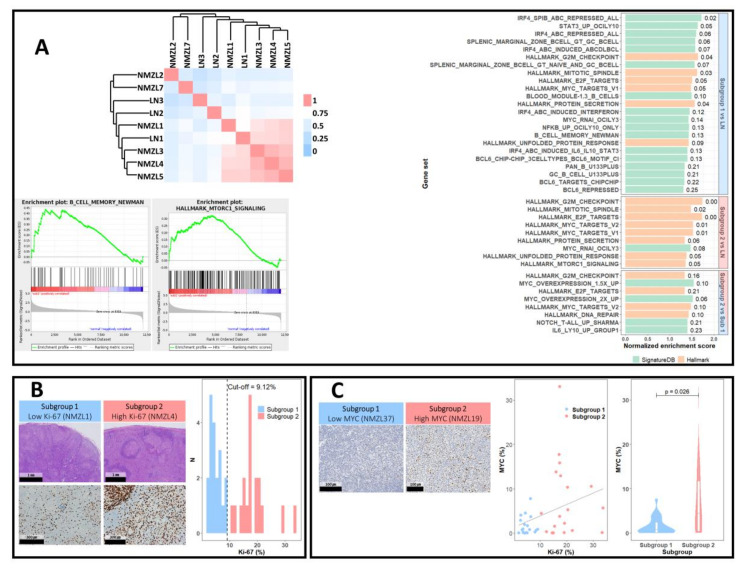
Subgroups of NMZL. (**A**) Pearson correlation matrix was created based on the GEPs of six NMZLs and three non-neoplastic LNs. An unsupervised clustering analysis revealed strong associations among NMZL3, 4, and 5, leading to determination of subgroup 1 (NMZL1, 2 and 7) and subgroup 2 (NMZL3, 4 and 5). GSEA between subgroup 1 and LNs and between subgroup 2 and LNs showed that certain gene sets were enriched in only one of the two subgroups. GSEA between subgroups 2 and 1 further supported the enrichment of cellular proliferation pathways and the MYC pathway in subgroup 2. (**B**) Ki-67 proliferation indices in the whole population was performed, and two subgroups were defined according to the median Ki-67 value. (**C**) Representative cases of MYC expression are shown. MYC positivity was assessed by digital image analysis and was 3.78% and 12.90% in NMZL37 (left) and NMZL19 (right), respectively. The scatter plot shows the association between Ki-67 proliferation index and MYC positivity, and the higher MYC positivity of subgroup 2 is shown as a violin plot.

Unsupervised clustering analysis was performed based on the results of a sample-to-sample Pearson correlation analysis (Figure 5A). While NMZL1, 2 and 7 (subgroup 1) were observed to be admixed with non-neoplastic LNs (LN1, 2 and 3), NMZL3, 4, and 5 (subgroup 2) were grouped together, with a significantly high correlation (Pearson’s *r* = 0.925, NMZL3 and 4; 0.899, NMZL3 and 5; 0.931, NMZL4 and 5). Thus, we hypothesized that subgroup 2 has unique characteristics and performed further GEP analyses of subgroups 1 and 2.

The gene sets enriched in the two subgroups compared with non-neoplastic LNs largely overlapped with those enriched in all six NMZLs (Figure 5A). However, a few newly noted gene sets were identified; a gene set representing the memory B-cell signature (B_CELL_MEMORY_NEWMAN) [16] was enriched only in subgroup 1 (NES = 1.43; FDR *q*-value = 0.13), whereas the MTORC1-signaling pathway (NES = 1.36; FDR *q*-value = 0.05) was enriched only in subgroup 2. We also performed GSEA between the two subgroups; compared with subgroup 1, gene sets related to the cell cycle, DNA repair, MYC-signaling, and NOTCH pathway were significantly enriched in subgroup 2, as well as a set of genes induced immediately by IL-6 stimulation in a DLBCL cell line. These findings further support the existence of NMZL subgroups with discrete characteristics.

The DEGs of subgroups compared with normal LNs were subjected to upstream regulator analysis by IPA, revealing that the subgroups were governed by discrete upstream regulators (Table S8 and Figure S3). Whereas POU2F2 and NUPR1 were common to both subgroups, subgroup 2—presumably a more proliferative subset of NMZLs based on the GSEA results—was regulated by a larger number of oncogenic molecules and TFs, including MYC, MITF, FOXM1, ATF6, E2F1, E2F3, and MYBL2.

### 2.6. NMZL Subgroups and Their Clinicopathological Implications

The GEP of the discovery set suggested that subgroup 2 NMZLs are characterized by an increased proliferative drive and MYC-signaling. Concordantly, we found a significantly higher Ki-67 labeling index in subgroup 2 compared with subgroup 1 (Figure 5B; *p* = 0.04953; Mann–Whitney test). Therefore, we classified all patients in the discovery and validation sets according to the Ki-67 proliferation index and assessed the status of the MYC pathway by IHC.

Image analyses of representative areas of the tumors (median number of analyzed nuclei, 12,453; range, 8650–28,840) were performed in all cases. Areas containing relatively monomorphic monocytoid B-cells were selected, and GCs were carefully excluded to prevent overestimation (Figure S4). The Ki-67 index ranged from 2.0% to 33.4%. Cases were classified into Ki-67^Low^ (subgroup 1) and Ki-67^High^ groups (subgroup 2) based on a cutoff median Ki-67 index of 9.1% (Figure 5B). Subgroup 2 was significantly associated with older age (*p* = 0.043), elevated lactate dehydrogenase (LDH) level (*p* = 0.002), advanced stage (*p* = 0.021), higher IPI score (*p* = 0.001) and progression events (*p* = 0.021) (Table 2). To assess whether the large cell component of the tumor was associated with the subgroups, we reviewed the histology of all samples and estimated the proportions of the large cell component (Figure S5 and Table S9). Subgroup 2 was associated with a higher proportion (≥20%) of the large cell component (Table 2; *p* = 0.009). These findings support the biological relevance of the NMZL subgrouping.

The percentage of MYC positivity quantified by image analysis ranged from 0.01% to 33.01% (Figure 5C). A significant positive correlation between MYC positivity and the Ki-67 proliferation index was observed (Spearman’s rho = 0.383, *p* = 0.018; Mann–Whitney *p* = 0.026) (Figure 5C). Using 10% as the cutoff IHC positivity rate for establishing the MYC^High^ and MYC^Low^ groups, we found significant associations between the MYC^High^ group and advanced stage (*p* = 0.029), and progression events (*p* = 0.002) (Table S10).

**Figure 6:**
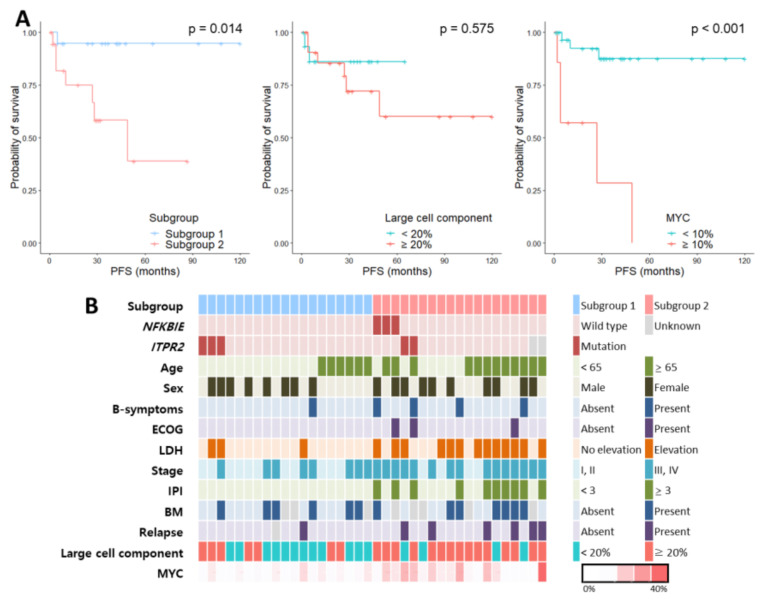
Summary of the clinicopathological characteristics. (**A**) Kaplan–Meier curves of progression-free survival (PFS) according to subgroups, large cell component, and MYC expression are shown. (**B**) The characteristics of the 38 NMZLs are summarized and depicted.

Progression-free survival (PFS) was analyzed according to various clinicopathological factors (Figure 6A and Figure S6); subgroup 2 was noted for its significant association with shorter PFS (*p* = 0.014), along with MYC^High^ (*p* < 0.001), elevated LDH level (*p* = 0.033) and advanced stage (*p* = 0.005). When patients treated with R-CVP based regimen and R-CHOP based regimen were separately analyzed, we observed similar trends of poor prognosis of subgroup 2, though statistical significances were not achieved (Figure S7). Collectively, these data suggest that NMZLs can be distinguished into clinically relevant subgroups according to the Ki-67 proliferation index (Figure 6B), which is significantly associated with MYC expression.

## 3. Discussion

There is little overlap among studies on the genetic features of NMZLs [6,7], possibly due to the heterogeneity of the disease and the lack of prior studies. By WES and RNA-seq on the discovery cohort, followed by validation in an extended cohort of patients with NMZLs, we identified novel genetic alterations, enriched cellular pathways, and clinically relevant subgroups.

Some genetic characteristics reported in previous studies on NMZL were reproduced here [6,7,8,10,17], including the high frequencies of mutations in genes affecting chromatin modifiers and BCR-signaling pathways and a lack of recurrent driver fusions. However, we did not find *PTPRD* or *BRAF* mutations, the most important genetic alterations in NMZLs reported in two WES-based studies [6,7]. We report the discovery of two novel recurrent genetic alterations in *NFKBIE* and *ITPR2* in NMZL (Figure 6); though we did not find significantly different clinicopathological features between patients harboring mutations of these genes and those do not, these novel findings are noteworthy for their potential to be used as diagnostic or therapeutic biomarkers.

*NFKBIE* encodes NFKB inhibitor epsilon (NFKBIE), a negative regulator of the NF-κB pathway [18]. The *NFKBIE* Y254fs mutation has been reported in aggressive chronic lymphocytic leukemia (CLL), Hodgkin lymphoma (HL), DLBCL, primary mediastinal B-cell lymphoma (PMBCL) and a few cases of low-grade B-cell NHL, including follicular lymphoma (FL), EMZL [19], and SMZL [11,20]. However, this mutation has never been reported in patients with NMZLs. In this study, the *NFKBIE* Y245fs mutation was observed in 7.9% (3/38) of patients, all in subgroup 2. These findings suggest a role for the NF-κB pathway in the pathogenesis of subgroup 2, which warrants further investigation.

Mutations in *ITPR2* have been reported in various types of solid tumors, including endometrial carcinoma (9.3%) [21], malignant melanoma (6.4%) [22], urothelial carcinoma (7.0%) [23], and rarely in lymphoid neoplasm including DLBCL (3.0–7.5%) [24,25], multiple myeloma (1.5%) [26], CLL (0.2%) [27], and a case with Sezary syndrome [28]. To our knowledge, NMZL has the highest frequency of *ITPR2* mutations, which were encountered in 13.9% of patients with NMZL. Notably, *ITPR2* mutations identified in NMZLs were clustered within the MIR domain, unlike other types of lymphoma. *ITPR2* encodes a member of the inositol 1,4,5-triphosphate receptor (IP_3_R) family, and the major function of ITPR2 in B-cells is BCR-induced Ca^2+^ signaling process by binding to IP_3_, leading to activation of the TFs that orchestrate diverse genetic programs. A study using a B-cell specific IP_3_R family triple-knockout mouse model demonstrated that impaired Ca^2+^ signaling results in abnormal B-cell development, and that intact IP_3_R function supports BCR-induced proliferation and survival of B-cells [29]. Although the functional consequences of *ITPR2* variants in NMZL are yet to be determined, these variants were identified within the MIR domain, which is a part of the IP_3-_binding core [30]. Thus, it is possible that *ITPR2* mutations in NMZL alter the ligand-binding ability of ITPR2. Together with the other genetic alterations involving BCR-signaling pathway, recurrent clustered *ITPR2* mutations may contribute to the pathogenesis of NMZLs.

Previous studies on SMZLs reported frequent *KLF2*, *NOTCH2*, *TRAF3*, *KMT2D*, and *TNFAIP3* mutations [31,32,33]. None of our patients harbored mutations in these genes, except for *KMT2D.* Characteristics genetic features of EMZLs included *TNFAIP2*, *CREBBP*, *TBL1XR1*, *MALT1* or *BCL10* alterations [34,35]. However, most of the above findings were not present in our patients; only one case (NMZL7) harbored the *TBL1XR1* V445G mutation, near the site reported in ocular EMZL [35]. These results support the notion that NMZL is an independent entity with a distinct pathogenesis.

The results of upstream regulator analyses showed that IRF4 was significantly inhibited in NMZLs, consistent with the GSEA results. IRF4 plays diverse context-specific roles at different stages of B-cell development [36]. A previous study using *IRF4*-knockout mice showed that IRF4 deficiency enhanced the activation of the *NOTCH2* pathway, which is critical for MZ differentiation, thereby causing aberrant accumulation of follicular B-cells in the MZ area [37]. We also found increased proliferative drive in NMZLs, which was caused by various oncogenic signaling pathways (e.g., MYC, NOTCH, NF-κB, and STAT3) and B-cell specific TFs (e.g., POU2F2). Therefore, the combined effects of aberrant MZ differentiation, caused by an altered IRF4-NOTCH2 axis, and additional oncogenic drive, may explain the lymphomagenesis of NMZL.

An unsupervised clustering analysis suggested the existence of a distinct subgroup (NMZL3, 4 and 5) from the other NMZL samples. Based on GSEA and IPA, subgroup 1 showed enrichment of memory B-cell signature; a previous microarray-based GEP analysis of NMZL also suggested that NMZL resembles normal MZ B-cells and memory B-cells [9]. Our data provide additional evidence for the postulated cell-of-origin of NMZLs. Subgroup 2 harnessed MTORC1-pathway, implying more active BCR-signaling, and a broader repertoire of oncogenic regulators.

With reference to additional proliferative pressure in subgroup 2 compared with subgroup 1, we defined both subgroups in the validation set according to the Ki-67 proliferative index. Digitally assessed Ki-67 labeling index showed a bimodal distribution, which enabled the two subgroups to be distinguished. An NMZL subset with an increased Ki-67 proliferation index was previously suggested [4], although the biological implications were unclear. We showed that subgroup 2 had a more aggressive clinical presentation and a significantly shorter PFS. Moreover, the higher expression of MYC in subgroup 2, in agreement with its association with MYC-regulated pathways, could be the mechanism underlying the distinct clinical presentation and prognosis of subgroup 2. Although MYC is a master regulator involved in the lymphomagenesis of diverse lymphoid tumors [38], few studies have reported its role in low-grade B-cell NHL. A series of EMZLs suggested that higher expression of MYC is associated with an elevated risk of high-grade transformation [39]. In accordance with this, we provide an evidence that MYC could be a key factor in high-risk subset NMZL.

The proper grading of NMZL based on histologic features has long been an unresolved issue. Nathwani et al. suggested that NMZL cases with large cells in more than 20% of the tumor are related to more aggressive behavior [40]; however, biological significance of the large cell component was unclear. In this study, cases with a large cell proportion more than 20% was associated with a higher Ki-67 (subgroup 2) but not related to MYC expression, PFS, and other clinicopathological features. Taken together, the findings suggest that categorizing NMZL based on Ki-67 index could be more clinically meaningful than a proportion of the large cell component.

Nevertheless, the different treatment approaches and underlying medical conditions of the patients may have had impact on the disease course. To specifically analyze whether the survival difference could be attributed to the subgroup regardless of the treatment strategies or co-morbidities, further validation studies on a larger number of samples are warranted. Due to the rarity of this disease, this study was performed on a small discovery set; however, we established an extended validation set to confirm our findings and strengthen the level of evidence. The careful review of histologic features was performed by experienced hematopathologists to confirm diagnosis. Though NMZL2 harbored *MYD88* L265P mutation, *CXCR4* mutation was not detected, histopathologic features were not typical for LPL, and the patient did not show any clinical evidence suggestive of LPL. Since *MYD88* L265P mutation alone does not meet the diagnostic criteria of LPL and considering that the mutation was also reported in a previous study on patients with NMZL^6^, we decided to include this case. Furthermore, the functional consequences of the novel mutations found in our study should be tested in the future to gain further insights into this disease.

In summary, using WES and RNA-seq, we identified novel mutations with potential biological implication and cellular pathways harnessed by NMZLs, and defined subgroups of this entity. Our findings enhance our understanding of NMZL and suggest that it is an independent disease entity with distinct genetic and transcriptional features.

## 4. Materials and Methods

### 4.1. Patients and Samples

The discovery set included eight patients with NMZL diagnosed at Seoul National University Hospital (SNUH) from 2014 to 2017, and the validation set consisted of 16 patients with NMZLs from SNUH and 14 patients with NMZL diagnosed at Seoul National University Bundang Hospital (SNUBH) from 2002 to 2018. All NMZL cases were diagnosed according to the revised 4th World Health Organization (WHO) classification [41]. All samples were acquired at the initial diagnosis, except NMZL7, as the initial sample of this patient failed the quality assessment for next-generation sequencing; therefore, the sample acquired at the progression was used; therefore, NMZL7 was excluded from all subsequence analyses regarding progression-related variables. Hematoxylin–eosin (H&E) stained slides of all cases with CD3, CD20, BCL2, BCL6, CD10, cyclin D1, CD5, CD21, CD23, MUM1 and Ki-67 immunostainings and EBV in situ hybridization were reviewed by five experienced hematopathologists (J.K., C.L., J.H.P., C.W.K. and Y.K.J.). Cases with evidence of extranodal disease were excluded, as were cases showing overt high-grade transformation. The large cell component was assessed as the proportion of large cells in the tumor area. The complete blood counts (CBC), BM examination, and serologic test results of all patients were also carefully reviewed to exclude chronic lymphocytic leukemia (CLL) and lymphoplasmacytic lymphoma (LPL). Clinicopathologic characteristics including serum LDH levels were retrieved from the medical records; according to the reference range values in the clinical laboratories in SNUH and SNUBH, patients who had serum LDH levels above 225 IU/L were regarded as having increased LDH levels. Formalin-fixed, paraffin-embedded (FFPE) tissue samples of discovery and validation sets were retrieved. Matched non-neoplastic samples from two patients in the discovery set, and thee non-related FFPE samples of non-neoplastic lymph nodes (LNs) were also included. This study was approved by the Institutional Review Boards (IRB) of SNUH (1809-143-977) and SNUBH (B-1306-208-301).

### 4.2. WES and Processing of Variants

Tumor genomic DNA from eight patients with NMZL from the discovery set and matched germline DNA from two of these patients (NMZL3 and NMZL5) were used to construct sequencing libraries using SureSelect^XT^ Human All Exon V5 (Agilent Technologies, Santa Clara, CA, USA). Sequencing was performed on the Illumina HiSeq platform with the paired-end 2 × 101 bp read option. All steps were performed at Theragene ETEX Bio Institute (Suwon, Korea).

Sequencing reads from tumor and germline samples were aligned to the reference human genome hg19/NCBI GRCh 37 using the Burrows-Wheeler Aligner (BWA) (v.0.7.12) [42], achieving a mean on-target depth of 106.21× with at least 75.3% of the target exome covered at 50×. Deduplication and local re-alignment were performed using Picard (v.1.92) and Genome Analysis Tool Kit (GATK, v.2.3-9) [43]. Single-nucleotide variants (SNV) were called using MuTect (v.1.1.4) [44], and short insertions and deletions (indels) were identified by indelocator (v.2.3-9). Variant annotation was performed using SnpEff (v.4.2). Stringent variant filtering was performed to reduce contamination by low-quality or germline variants, as described in the Supplementary information.

Visual inspection was carried out in 10 selected genes previously reported to exhibit structural variation in B-cell NHLs: *BCL2*, *BCL3*, *BCL6*, *BCL10*, *BIRC3*, *CCND1*, *FOXP1*, *MALT1*, *C-MYC*, and *PAX5* (Table S11). Aligned reads were visually inspected using Integrative Genomics Viewer (IGV), and we tried to identify the regions that had at least one read containing a possible fusion breakpoint, or in which the mate pair was aligned with over a 10,000-bp gap or mapped to a different chromosome.

### 4.3. RNA-seq and Gene Expression Analysis

RNA-seq was performed on NMZL cases in the discovery set and three non-related non-neoplastic LN samples. Sequencing libraries were prepared using the TruSeq^TM^ RNA Exome kit, and sequencing was performed on the Illumina HiSeq platform at Theragene ETEX Bio Institute (Suwon, Korea) using the paired-end 2 × 100 bp option for tumor samples and the 2 × 150 bp option for normal samples.

Pearson correlation coefficients between transcripts per million (TPMs) of genes from each sample were calculated to estimate sample-to-sample correlations. Genes other than those with a TPM ≥ 1.0 in more than three NMZL tumor samples were filtered out; however, genes with a TPM ≥ 1.0 in all three normal LN samples were included. Differentially expressed genes (DEGs) between the NMZL and normal LN samples were defined as those with a log_2_ fold-change (FC) value >2.0 or <−2.0. Gene set enrichment analysis (GSEA) by gene set permutation [45], and knowledge-based network analysis by Ingenuity Pathway Analysis (IPA^®^, Qiagen, Hilden, Germany, Spring 2019 Release) [46] were performed. The details are described in the Supplementary Information.

### 4.4. Direct Sequencing and Immunohistochemistry (IHC)

Direct sequencing of *NFKBIE*, *ITPR2*, *CD70*, *CD79B* and *CARD11* was conducted (Table S12). To validate gene expression data, IHC analyses of IRF4 (MUM1), MYC, Ki-67 and CD70 were performed on 4-μm-thick whole sections of FFPE tissue samples and assessed manually or using image analyzer. The details are described in the Supplementary Information.

### 4.5. Statistical Analysis

Chi-squared, linear-by-linear and Fisher’s exact tests were performed to compare categorical variables, and the Mann–Whitney test to compare continuous variables. The log-rank test was used for survival analysis. The false discovery rate (FDR) for GSEA was calculated using the gene permutation version of multiple hypothesis testing method [45]. Statistical significance was defined as *p* < 0.05 or *q* < 0.25. Statistical analysis was performed using the R statistical package 3.6.0 (http://www.r-project.org).

## 5. Conclusions

We found that *NFKBIE* 4-bp deletion and *ITPR2* mutations affecting the MIR domain are novel genetic alterations of NMZL. The transcriptomic profiles of NMZL suggested the presence of subgroups distinguished by proliferative activity, which appear to have distinct clinical, histopathological and genetic features.

## Figures and Tables

**Table 1:** Clinicopathological features of study population.

		Discovery Set	Validation Set	Total	*p*
Age	Median (min–max)	64 (42–78)	63 (29–85)	64 (29–85)	0.902
	<60 years	4 (50.0%)	15 (50.0%)	19 (50.0%)	1.000
	≥60 years	4 (50.0%)	15 (50.0%)	19 (50.0%)	
Sex	Male	4 (50.0%)	16 (53.3%)	20 (52.6%)	1.000
	Female	4 (50.0%)	14 (46.7%)	18 (47.4%)	
B-symptoms	Absent	7 (87.5%)	26 (86.7%)	33 (86.8%)	1.000
	Present	1 (12.5%)	4 (13.3%)	5 (13.2%)	
ECOG PS	0	5 (62.5%)	15 (53.6%)	20 (55.6%)	0.454
	1	3 (37.5%)	10 (35.7%)	13 (36.1%)	
	2	0 (0.0%)	3 (10.7%)	3 (8.3%))	
LDH	No elevation	4 (50.0%)	16 (57.1%)	20 (55.6%)	1.000
	Elevation	4 (50.0%)	12 (42.9%)	16 (44.4%)	
Ann Arbor Stage	I	1 (12.5%)	5 (16.7%)	6 (15.8%)	0.250
	II	0 (0.0%)	9 (30.0%)	9 (23.7%)	
	III	3 (37.5%)	5 (16.7%)	8 (21.1%)	
	IV	4 (50.0%)	11 (36.7%)	15 (39.5%)	
IPI	0	0 (0.0%)	6 (21.4%)	6 (16.7%)	0.353
	1	2 (25.0%)	7 (25.0%)	9 (25.0%)	
	2	4 (50.0%)	7 (25.0%)	11 (30.6%)	
	3	1 (12.5%)	6 (21.4%)	7 (19.4%)	
	4	1 (12.5%)	2 (7.1%)	3 (8.3%)	
BM involvement	Absent	4 (50.0%)	13 (59.1%)	17 (56.7%)	0.698
	Present	4 (50.0%)	9 (40.9%)	13 (43.3%)	
Progression	No	5 (62.5%)	25 (83.3%)	30 (78.9%)	0.327
	Yes	3 (37.5%)	5 (16.7%)	8 (21.1%)	
HBV or HCV infection	Absent	5 (62.5%)	26 (86.7%)	31 (81.6%)	0.146
	Present	3 (37.5%)	4 (13.3%)	7 (18.4%)	
Large cell component	<20%	4 (50.0%)	12 (40.0%)	16 (42.1%)	0.698
	≥20%	4 (50.0%)	18 (60.0%)	22 (57.9%)	
Total		8 (21.1%)	30 (78.9%)	38 (100.0%)	

Bone marrow (BM); Eastern Cooperative Oncology Group performance status (ECOG PS); International Prognostic Index (IPI); lactate dehydrogenase (LDH); hepatitis B virus (HBV); hepatitis C virus (HCV).

**Table 2:** Clinicopathological characteristics according to subgroup determined by the Ki-67 proliferation index.

		Subgroup 1 (Ki-67^Low^)	Subgroup 2 (Ki-67^High^)	Total	*p*
Age	Median (range, years)	58 (29–79)	70 (30–85)	64 (29–85)	0.043
	<60 years	13 (68.4%)	6 (31.6%)	19 (50.0%)	0.050
	≥60 years	6 (31.6%)	13 (68.4%)	19 (50.0%)	
Sex	Male	11 (57.9%)	9 (47.4%)	20 (52.6%)	0.516
	Female	8 (42.1%)	10 (52.6%)	18 (47.4%)	
B-symptoms	Absent	18 (94.7%)	15 (78.9%)	33 (86.8%)	0.340
	Present	1 (5.3%)	4 (21.1%)	5 (13.2%)	
ECOG PS	0	13 (72.2%)	7 (38.9%)	20 (55.6%)	0.022
	1	5 (27.8%)	8 (44.4%)	13 (36.1%)	
	2	0 (0.0%)	3 (16.7%)	3 (8.1%)	
LDH	No elevation	14 (82.4%)	6 (31.6%)	20 (55.6%)	0.002
	Elevation	3 (17.6%)	13 (68.4%)	16 (44.4%)	
Ann Arbor Stage	I	6 (31.6%)	0 (0.0%)	6 (15.8%)	0.021
	II	5 (26.3%)	4 (21.1%)	9 (23.7%)	
	III	2 (10.5%)	6 (31.6%)	8 (21.1%)	
	IV	6 (31.6%)	9 (47.4%)	15 (39.5%)	
IPI	0	5 (29.4%)	1 (5.3%)	6 (16.7%)	0.001
	1	6 (35.3%)	3 (15.8%)	9 (25.0%)	
	2	6 (35.3%)	5 (26.3%)	11 (30.6%)	
	3	0 (0.0%)	7 (36.8%)	7 (19.4%)	
	4	0 (0.0%)	3 (15.8%)	3 (8.3%)	
BM involvement	Absent	10 (62.5%)	7 (50.0%)	17 (56.7%)	0.491
	Present	6 (37.5%)	7 (50.0%)	13 (43.3%)	
HBV or HCV infection	Absent	17 (89.5%)	14 (73.7%)	31 (81.6%)	0.209
	Present	2 (10.5%)	5 (26.3%)	7 (18.4%)	
Progression	No	17 (94.4%)	12 (63.2%)	29 (78.4%)	0.021
	Yes	1 (5.6%)	7 (36.8%)	8 (21.6%)	
*NFKBIE*	Wild type	19 (100.0%)	16 (84.2%)	35 (92.1%)	0.230
	Mutant	0 (0.0%)	3 (15.8%)	3 (7.9%)	
*ITPR2*	Wild type	16 (84.2%)	15 (88.2%)	31 (86.1%)	1.000
	Mutant	3 (15.8%)	2 (11.8%)	5 (13.9%)	
MYC IHC	<10%	19 (100.0%)	12 (63.2%)	31 (81.6%)	0.008
	≥10%	0 (0.0%)	7 (36.8%)	7 (18.4%)	
Large cell component	<20%	12 (63.2%)	4 (21.1%)	16 (42.1%)	0.009
	≥20%	7 (36.8%)	15 (78.9%)	22 (57.9%)	
Total		19 (50.0%)	19 (50.0%)	38 (100.0%)	

Bone marrow (BM); Eastern Cooperative Oncology Group performance status (ECOG PS); International Prognostic Index (IPI); lactate dehydrogenase (LDH); hepatitis B virus (HBV); hepatitis C virus (HCV); immunohistochemistry (IHC).

## Supplementary Materials

The following are available online at https://www.mdpi.com/2072-6694/12/6/1669/s1, Figure S1: Histopathologic characteristics of representative cases, Figure S2: The upstream regulators MITF and KDM5B and their predicted networks in NMZL, Figure S3: Network analysis of each subgroup of NMZL, Figure S4: Quantification of the Ki-67 proliferation index by image analysis, Figure S5: Representative cases for estimation of the large cell component. Figure S6. Progression-free survival according to various clinicopathological factors, Figure S7: Separate survival analysis within patients treated with either R-CVP based or R-CHOP based regimen, Table S1: Detailed description of clinicopathological features of the study population, Table S2: Mutations in the discovery set identified by whole exome sequencing, Table S3: Clinicopathological information according to *NFKBIE* status, Table S4: In silico prediction of *ITPR2* mutations, Table S5: Clinicopathological features according to *ITPR2* status, Table S6: Upstream regulators of genes differentially expressed in nodal marginal zone lymphoma samples, Table S7: Results of regulator effector analysis and consistency scores, Table S8: Upstream regulators of genes differentially expressed among subgroups of nodal marginal zone lymphoma, Table S9: Clinicopathological features according to the large cell component, Table S10: Clinicopathological features according to MYC positivity, Table S11: List of common fusion genes in B-cell non-Hodgkin lymphoma, Table S12: Polymerase chain reaction primers and experimental conditions.
