# Supplementary material for: Discovery of Novel Recurrent Mutations and Clinically Meaningful Subgroups in Nodal Marginal Zone Lymphoma
Source: Cancers (Basel). 2020 Jun 23;12(6):1669. doi: 10.3390/cancers12061669 (PMC7352856; doi:10.3390/cancers12061669)
Supplement: Supplementary file 1 [file cancers-12-01669-s001.zip › NMZL_Supplementary Information_Koh et al_Cancers_rev.pdf]

Supplementary Information

Discovery of novel recurrent mutations and clinically meaningful subgroups in nodal marginal zone lymphoma

Jiwon Koh[1-2*], Insoon Jang[3*], Seongmin Choi[3-4*], Sehui Kim[1-2], Ingeon Jang[2], Hyun Kyung Ahn[5], Cheol Lee[1-2], Jin Ho Paik[6], Chul Woo Kim[1-2], Megan S. Lim[7], Kwangsoo Kim[3†], Yoon Kyung Jeon[1-2,5†]

[1] Department of Pathology, Seoul National University Hospital, Seoul, Republic of Korea

[2] Department of Pathology, Seoul National University College of Medicine, Seoul, Republic of Korea

[3] Division of Clinical Bioinformatics, Biomedical Research Institute, Seoul National University Hospital, Seoul, Republic of Korea

[4] Seoul National University College of Medicine, Seoul, Republic of Korea

[5] Cancer Research Institute, Seoul National University, Seoul, Republic of Korea

[6] Department of Pathology, Seoul National University Bundang Hospital, Seongnam-si, Republic of Korea

[7] Department of Pathology and Laboratory Medicine, University of Pennsylvania Perelman School of Medicine, Philadelphia, PA, USA

*These authors contributed equally to this work

Supplementary Methods

Supplementary References

Supplementary Figure Legends

Supplementary Methods

Whole exome sequencing (WES)

Tumor genomic DNA from eight NMZL cases in the discovery set and matched germline DNA from two cases (NMZL3 and NMZL5) were extracted and quantified using the Qubit fluorometer (Invitrogen, Eugene, OR, USA). Sequencing libraries were prepared using SureSelect^{XT} Human All Exon V5 (Agilent Technologies, Santa Clara, CA, USA), and sequencing was performed on the Illumina HiSeq platform using the paired-end 2×101 bp read option following the manufacturer's instructions. Exome library preparation and high-throughput sequencing were performed at Theragene ETEX Bio Institute (Suwon, Republic of Korea).

Sequence mapping, variant calling and filtering

Sequencing reads from tumor and germline samples were aligned to the reference human genome hg19/NCBI GRCh 37 using Burrows-Wheeler Aligner (BWA) (v.0.7.12) [1], achieving a mean on-target depth of 106.21× with at least 75.3% of the target exome covered at 50×. Deduplication and local re-alignment were performed using Picard (v1.92) and Genome Analysis Tool Kit (GATK, v2.3-9) [2]. Single-nucleotide variants (SNV) were called using MuTect (v1.1.4) [3], and short insertions and deletions (indels) were identified by indelocator (v2.3-9). Variant annotation was performed using SnpEff (v.4.2). The initial set of variants was filtered by removing synonymous variants, low-quality variants, and variants with a minor allele frequency (MAF) ≥ 0.1% in the Korean Reference Genome Database (KRGDB), variants with MAF ≥ 0.01% in ExAC East Asian (ExAC_EAS) or gnomAD East

Asian (gnomAD_EAS) databases. Single-nucleotide variants (SNVs) and indels with a variant allele frequency (VAF) of < 5%, or mapped read count ≤ 10 were filtered out. Gene with a large size, very low expression and/or very late replications times (including *MUC16, MUC5B*, *TTN* and *PCLO)* were removed [4].

For six cases that lacked a matched normal control, stringent filtering was performed to reduce contamination by germline variants. An unmatched panel of normal (PoN) was generated by combining sequencing data from two non-malignant tissue samples from NMZL3 and NMZL5 in the discovery set with the in-house WES results of from thee additional non-neoplastic tissue samples from patients with other types of lymphoma. Using this PoN as a control for six tumor-only cases, any variants found in both the discovery set and PoN were considered to be germline and were discarded. For variants with available functional prediction results, additional filtering was performed by including only the variants assumed to have damaging effect on the protein function, i.e., nonsense, frameshift mutations, and mutations predicted to have a deleterious effect by the majority (allowing at most only one disagreement from three or more assessments) of variant impact prediction tools (PolyPhen-2 HVAR, PolyPhen-2 HDIV, SIFT, MutationTaster, and LRT). Missense mutations with no such available data on protein function were accepted. In addition, variants with a VAF of 45 – 55% or >70% were excluded to reduce germline contamination.

RNA-seq

Messenger RNA (mRNA) sequencing was performed for NMZL cases in the discovery set and for three non-related non-neoplastic LN samples (LN1, LN2 and LN3) from healthy individuals. Sequencing libraries were prepared using the TruSeq™ RNA

Exome kit, and transcriptome sequencing was performed on the Illumina HiSeq platform at Theragene ETEX Bio Institute (Suwon, Republic of Korea), using the paired-end 2 × 100 bp option for tumor samples and the 2 × 150 bp option for normal samples following the manufacturer's instructions. Raw FASTQ files were checked using FastQC (v.0.11.5) for quality assessment. NMZL6 and NMZL8 showed high duplication rates and low sequencing yields and thus were excluded from subsequent analyses.

Gene expression analysis

Adapter sequences in RNA-Seq reads were trimmed by Trimmomatic (v.0.36) [5], and filtered reads were aligned to the reference human genome using STAR aligner (v.2.6.0a) [6]. Aligned reads were quantified by RSEM (v.1.3.1) [7] as transcripts per million (TPM). The GRCh37/hg19 fasta file from the UCSC genome browser archive and the annotation gtf file from the UCSC refFlat table were used to create STAR and RSEM genomic indices. The Pearson correlation coefficients between the $\log_2$-transformed TPMs of genes were calculated to estimate the sample-to-sample correlation.

Genes other than those with a TPM ≥ 1.0 in more than three NMZL tumor samples were filtered out; however, genes with a TPM ≥ 1.0 in all of the three normal LN samples were included. Differentially expressed genes (DEGs) between NMZL and normal LN samples were defined as those with $\log_2$ fold-change (FC) values greater than 2.0 or less than -2.0. Gene set enrichment analysis (GSEA) [8] was carried out by gene set permutation on filtered genes using the gene sets from MSigDB (http://software.broadinstitute.org/gsea/msigdb) and the SignatureDB collection

(https://lymphochip.nih.gov/signaturedb/) [9]. A cut-off false discovery rate (FDR) q-value of ≤ 0.25 was applied to select significantly enriched signaling pathways.

Network analysis

We performed a knowledge-based network analysis using Ingenuity Pathway Analysis (IPA®, Qiagen, Hilden, Germany, Spring 2019 Release) [10] using DEGs defined by the absolute FC differences between NMZLs and non-neoplastic LNs. Upstream regulator analysis was used to identify possible upstream transcriptional regulators (transcription factors, enzymes, kinases or groups of molecules) involved in NMZLs. For prediction of upstream regulators, the p-value of overlap between DEGs in the input dataset and regulated genes listed in the Ingenuity Knowledge Base (IKB) was calculated by Fisher's exact test. In addition, the *z*-score was calculated using the formula below, which considers both the weight (w_i) and direction (x_i) of the upstream regulator effect.

$$z = \frac{x}{\sigma_x} = \frac{\sum_i w_i x_i}{\sqrt{\sum_i w_i^2}}$$

Following the manufacturer's recommendations, i) a p-value of overlap ≤ 0.01 calculated by Fisher's exact test and ii) a prediction *z*-score level of either ≥ 2 (activation) or ≤ -2 (inhibition) were used as cut-offs for determining the significance of upstream regulator analysis.

Similarly, regulator effector analysis was performed to determine biologic effects or diseases that can be predicted and expected by changes in the expression of upstream regulators. Both predicted regulator effects and observed gene expression patterns were

considered in this analysis. The usefulness of each statistically significant combination of regulators for predicting biological effects was evaluated by calculating the consistency score using the formula below (Pc, total number of consistent paths from regulator to function; Wc, weight for consistent paths, set to 1.0; Pi, total number of inconsistent paths; Wi, weight for inconsistent paths, set to -1.5; Pn, total number of non-causal paths; Wn, weight for non-causal paths, set to 0.0; S, total number of dataset targets; Ws, penalty weight for size of the network, set to 0.5):

$$\text{Consistency Score} = \frac{P_c \cdot W_c + P_i \cdot W_i + P_n \cdot W_n}{(S)^{W_s}}$$

Direct sequencing

To validate our findings, we performed direct sequencing to detect selected variants identified by WES. Genomic DNA was extracted using the Maxwell 16 FFPE Plus Tissue LEV DNA Purification kit (Promega, Madison, WI, USA) from 10 μm-thick sections prepared from FFPE tissue samples. Polymerase chain reaction (PCR) primers were designed using Primer-BLAST [11], and their sequences and the PCR conditions for each gene are listed in **Supplementary Table S2**. Nested PCR was performed using Ex *Taq* (TaKaRa Bio, Shiga, Japan). The PCR products were purified and subjected to direct sequencing using the ABI3730xl DNA analyzer (Applied Biosystems, Waltham, MA, USA).

Immunohistochemistry (IHC)

IHC was performed on 4-μm-thick whole sections of FFPE tissue samples.

Immunostaining for CD3 (F7.2.38, Ventana Medical Systems, Tucson, AZ, USA), BCL2 (124, Dako, Glostrup, Denmark), BCL6 (LN22, Novocastra, Newcastle Upon Tyne, UK), CD10 (56C6, Novocastra), IRF4 (MUM1p, Dako), MYC (EP121, Cell Marque, Rocklin, CA, USA), cyclin D1 (SP4, Ventana Medical Systems), CD5 (SP19, Ventana Medical Systems), Ki-67 (MIB-1, Dako), and CD70 (PA5-32700, Invitrogen) was performed using the BenchMark XT Autostainer (Ventana Medical Systems). Immunostaining for CD20 (L26, Dako), CD21 (2G9, Novocastra), and CD23 (SP23, Thermo Fisher Scientific, Waltham, MA, USA) was performed using the Bond-Max Autostainer (Leica Biosystems, Wetzlar, Germany).

Quantification of Ki-67- and MYC-positive cells by image analysis

All Ki-67 and MYC immunostained slides were digitally scanned using the Aperio AT2 (Leica Biosystems). After selection of a representative area of tumor cells, Ki-67- and MYC-positive cells were enumerated using the Nuclear V9 algorithm of ImageScope software (Aperio Technologies, Vista, CA, USA). The Ki-67 proliferation index and MYC-positivity were defined as the percentage of cells positive for the corresponding marker in the selected area.

Statistical analysis

Chi-squared, linear-by-linear and Fisher’s exact tests were performed to compare categorical variables, and the Mann-Whitney test to compare continuous variables. The log-rank test was used for survival analysis. The false discovery rate (FDR) for GSEA was

calculated using the gene permutation version of the multiple hypothesis testing method [8]. Statistical analysis was performed using the R statistical package v.3.6.0 (http://www.r-project.org).

Data availability

WES and RNA-seq datasets are deposited in European Genome Phenome Archive under accession number of EGAS00001003940.

Supplementary References

1. Li, H.; Durbin, R. Fast and accurate short read alignment with Burrows-Wheeler transform. *Bioinformatics* **2009**, *25*, 1754–1760.

2. McKenna, A.; Hanna, M.; Banks, E.; Sivachenko, A.; Cibulskis, K.; Kernytsky, A.; Garimella, K.; Altshuler, D.; Gabriel, S.; Daly, M.; et al. A. The Genome Analysis Toolkit: A MapReduce framework for analyzing next-generation DNA sequencing data. *Genome Res* **2010**, *20*, 1297–1303.

3. Cibulskis, K.; Lawrence, M. S.; Carter, S. L.; Sivachenko, A.; Jaffe, D.; Sougnez, C.; Gabriel, S.; Meyerson, M.; Lander, E. S.; Getz, G. Sensitive detection of somatic point mutations in impure and heterogeneous cancer samples. *Nat Biotechnol.* **2013**, *31*, 213–219.

4. Lawrence, M. S.; Stojanov, P.; Polak, P.; Kryukov, G. V.; Cibulskis, K.; Sivachenko, A.; Carter, S. L.; Stewart, C.; Mermel, C. H.; Roberts, S. A.; et al. Mutational heterogeneity in cancer and the search for new cancer-associated genes. *Nature* **2013**, *499*, 214–218.

5. Bolger, A. M.; Lohse, M.; Usadel, B. Trimmomatic: a flexible trimmer for Illumina sequence data. *Bioinformatics* **2014**, *30*, 2114–2120.

6. Dobin, A.; Davis, C. A.; Schlesinger, F.; Drenkow, J.; Zaleski, C.; Jha, S.; Batut, P.; Chaisson, M.; Gingeras, T. R. STAR: ultrafast universal RNA-seq aligner. *Bioinformatics* **2012**, *29*, 15–21.

7. Li, B.; Dewey, C. N. RSEM: accurate transcript quantification from RNA-Seq data with or without a reference. *BMC Bioinformatics* **2011**, *12*, 1–16.

8. Subramanian, A.; Tamayo, P.; Mootha, V. K.; Mukherjee, S.; Ebert, B. L.; Gillette, M. A.; Paulovich, A.; Pomeroy, S. L.; Golub, T. R.; Lander, E. S.; et al. Gene set enrichment analysis: a knowledge-based approach for interpreting genome-wide expression profiles. *Proc Natl Acad Sci USA.* **2005**, *102*, 15545–15550.

9. Shaffer, A. L.; Wright, G.; Yang, L.; Powell, J.; Ngo, V.; Lamy, L.; Lam, L. T.; Davis, R. E.; Staudt, L. M. A library of gene expression signatures to illuminate normal and pathological lymphoid biology. *Immunol Rev.* **2006**, *210*, 67–85.
10. Krämer, A.; Green, J.; Pollard, J., Jr; Tugendreich, S. Causal analysis approaches in Ingenuity Pathway Analysis. *Bioinformatics* **2013**, *30*, 523–530.
11. Ye, J.; Coulouris, G.; Zaretskaya, I.; Cutcutache, I.; Rozen, S.; Madden, T. L. Primer-BLAST: a tool to design target-specific primers for polymerase chain reaction. *BMC Bioinformatics* **2012**, *13*, 134.

Supplementary Figure Legends

Figure S1. Histopathologic characteristics of representative cases.

(A) NMZL2: a 56-year-old female underwent excisional biopsy of an enlarged cervical LN, which was replaced by diffuse proliferation of small, centrocyte-like, CD20-positive B-cells. A vaguely nodular pattern was detected by CD3 immunostaining, and the tumor cells were BCL2-positive, CD10-negative, and cyclin D1-negative and had a low Ki-67 proliferation index. CD23-positive follicular dendritic cells within residual germinal centers were also observed. **(B)** NMZL4: a 76-year-old female presented with a neck mass, which was composed of nodular lymphoid proliferation accompanied by prominent marginal zone hyperplasia (arrow). Follicles were surrounded by intermingled small and large lymphoid cells, some exhibiting blastic features. This case was categorized as having high Ki-67 proliferation. **(C)** NMZL5: a 64-year-old male patient presented with flank pain; a subsequent imaging work-up revealed systemic lymphadenopathy without involvement of extranodal sites. Biopsy of the supraclavicular LN showed small-sized B-cell non-Hodgkin lymphoma with a BCL2-positive, CD10-negative, CD23-negative and cyclin D1-negative immunophenotype, which is consistent with NMZL. **(D)** NMZL6: the cervical LN of a 68-year-old male was composed of diffuse and nodular growths of small-sized, low-proliferating, lymphoid cells with a rim of pale cytoplasm, causing nodal structural effacement and marked regression of germinal centers (marked as 'GC'). **(E)** NMZL7: the neck LN of a 64-year old male patient was composed of small CD20-positive, BCL2-positive, CD10-negative, and low-proliferating B-cells replaced with extracapsular extension. (Abbreviation: HE, hematoxylin and eosin)

Figure S2. The upstream regulators MITF and KDM5B and their predicted networks in NMZL.

MITF and KDM5B were predicted to be significant upstream regulators of NMZL and to interact with various transcription regulators, enzymes, kinases, transporters and transmembrane receptors.

Figure S3. Network analysis of each subgroup of NMZL.

(A) Upstream regulator analysis and regulator effect analysis predicted that regulation by POU2F2, a B-cell specific transcription factor, led to activation and inhibition of various molecules in subgroup 1, resulting in hematopoietic neoplasm. **(B)** Subgroup 2 was distinct from subgroup 1 in that a larger repertoire of transcription factors – including MYC, FOXM1 and ATF6 – was predicted as significant, the predicted steps of led to lymphohematopoietic neoplasia.

Figure S4. Quantification of the Ki-67 proliferation index by image analysis.

(A) When determining the Ki-67 proliferation index in a case (NMZL28) with residual, partially regressed germinal centers (GCs), we carefully excluded the GC areas to prevent overestimation. **(B)** In a case (NMZL38) with nodal architecture effacement without residual GC-like structures, image analyses were performed by selecting areas composed of diffuse proliferation of monocytoid B-cells.

Figure S5. Representative cases for estimation of the large-cell component.

(**A**) NMZL22 showed a nodular structure with a prominent marginal zone, where large-cell component was estimated as 5%. (**B**) NMZL23 showed marked marginal zone hyperplasia, 40% of which showed abundant large monocytoid cells.

Figure S6. Progression-free survival according to various clinicopathological factors.

Figure S7. Separate survival analysis within patients treated with either R-CVP based or R-CHOP based regimen
