# Supplementary material for: Discovery of Novel Recurrent Mutations and Clinically Meaningful Subgroups in Nodal Marginal Zone Lymphoma
Source: Cancers (Basel). 2020 Jun 23;12(6):1669. doi: 10.3390/cancers12061669 (PMC7352856; doi:10.3390/cancers12061669)

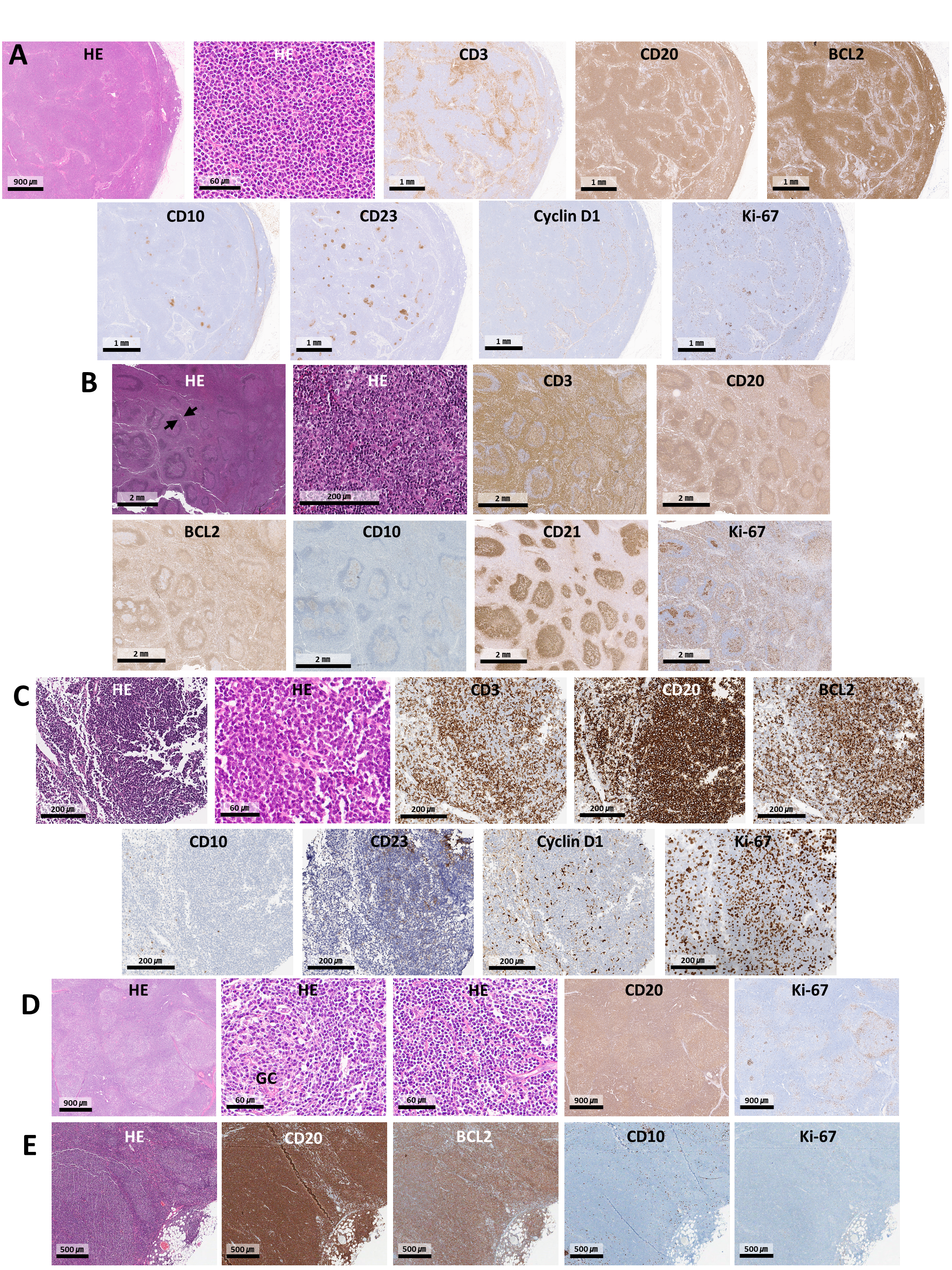


Figure S1. Histopathologic characteristics of representative cases.

(A) NMZL2: a 56-year-old female underwent excisional biopsy of an enlarged cervical LN, which was replaced by diffuse proliferation of small, centrocyte-like, CD20-positive B-cells. A vaguely nodular pattern was detected by CD3 immunostaining, and the tumor cells were BCL2-positive, CD10-negative, and cyclin D1-negative and had a low Ki-67 proliferation index. CD23-positive follicular dendritic cells within residual germinal centers were also observed. (B) NMZL4: a 76-year-old female presented with a neck mass, which was composed of nodular lymphoid proliferation accompanied by prominent marginal zone hyperplasia (arrow). Follicles were surrounded by intermingled small and large lymphoid cells, some exhibiting blastic features. This case was categorized as having high Ki-67 proliferation. (C) NMZL5: a 64-year-old male patient presented with flank pain; a subsequent imaging work-up revealed systemic lymphadenopathy without involvement of extranodal sites. Biopsy of the supraclavicular LN showed small-sized B-cell non-Hodgkin lymphoma with a BCL2-positive, CD10-negative, CD23-negative and cyclin D1-negative immunophenotype, which is consistent with NMZL. (D) NMZL6: the cervical LN of a 68-year-old male was composed of diffuse and nodular growths of small-sized, low-proliferating, lymphoid cells with a rim of pale cytoplasm, causing nodal structural effacement and marked regression of germinal centers (marked as ‘GC’). (E) NMZL7: the neck LN of a 64-year old male patient was composed of small CD20-positive, BCL2-positive, CD10-negative, and low-proliferating B-cells replaced with extracapsular extension. (Abbreviation: HE, hematoxylin and eosin)


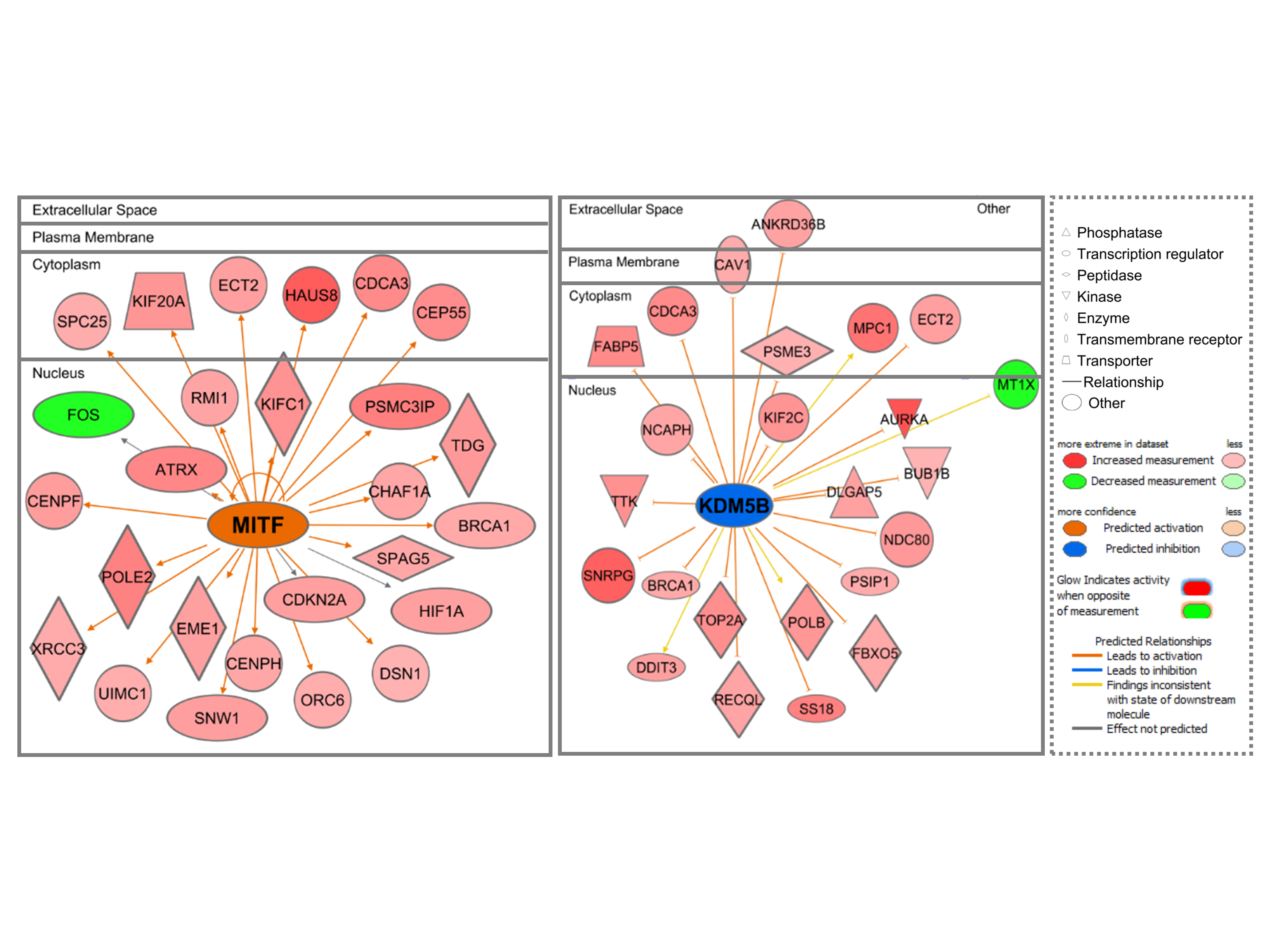


Figure S2. The upstream regulators MITF and KDM5B and their predicted networks in NMZL.

MITF and KDM5B were predicted to be significant upstream regulators of NMZL and to interact with various transcription regulators, enzymes, kinases, transporters and transmembrane receptors.


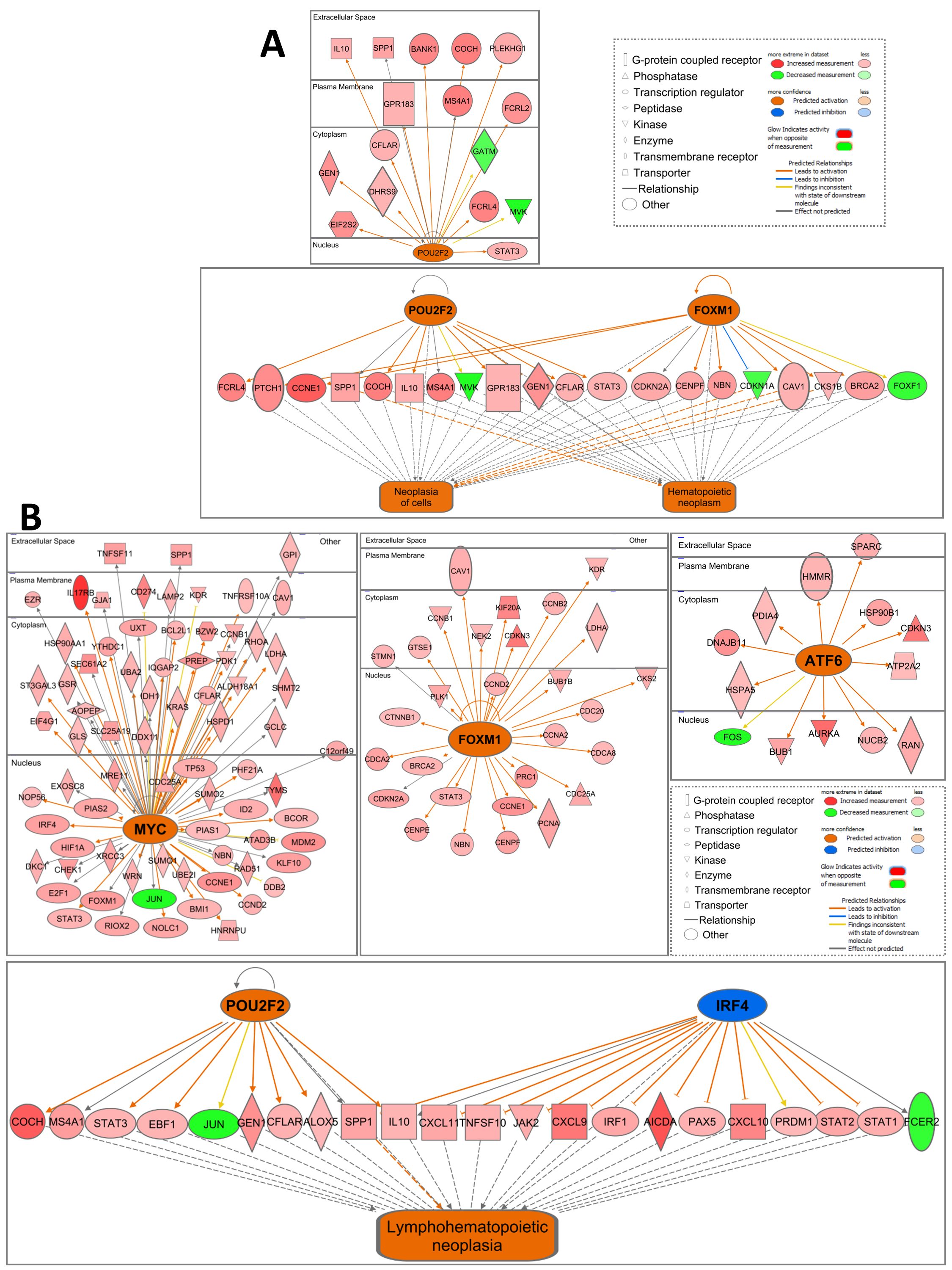


Figure S3. Network analysis of each subgroup of NMZL.

(A) Upstream regulator analysis and regulator effect analysis predicted that regulation by POU2F2, a B-cell specific transcription factor, led to activation and inhibition of various molecules in subgroup 1, resulting in hematopoietic neoplasm. (B) Subgroup 2 was distinct from subgroup 1 in that a larger repertoire of transcription factors – including MYC, FOXM1 and ATF6 – was predicted as significant, the predicted steps of led to lymphohematopoietic neoplasia.


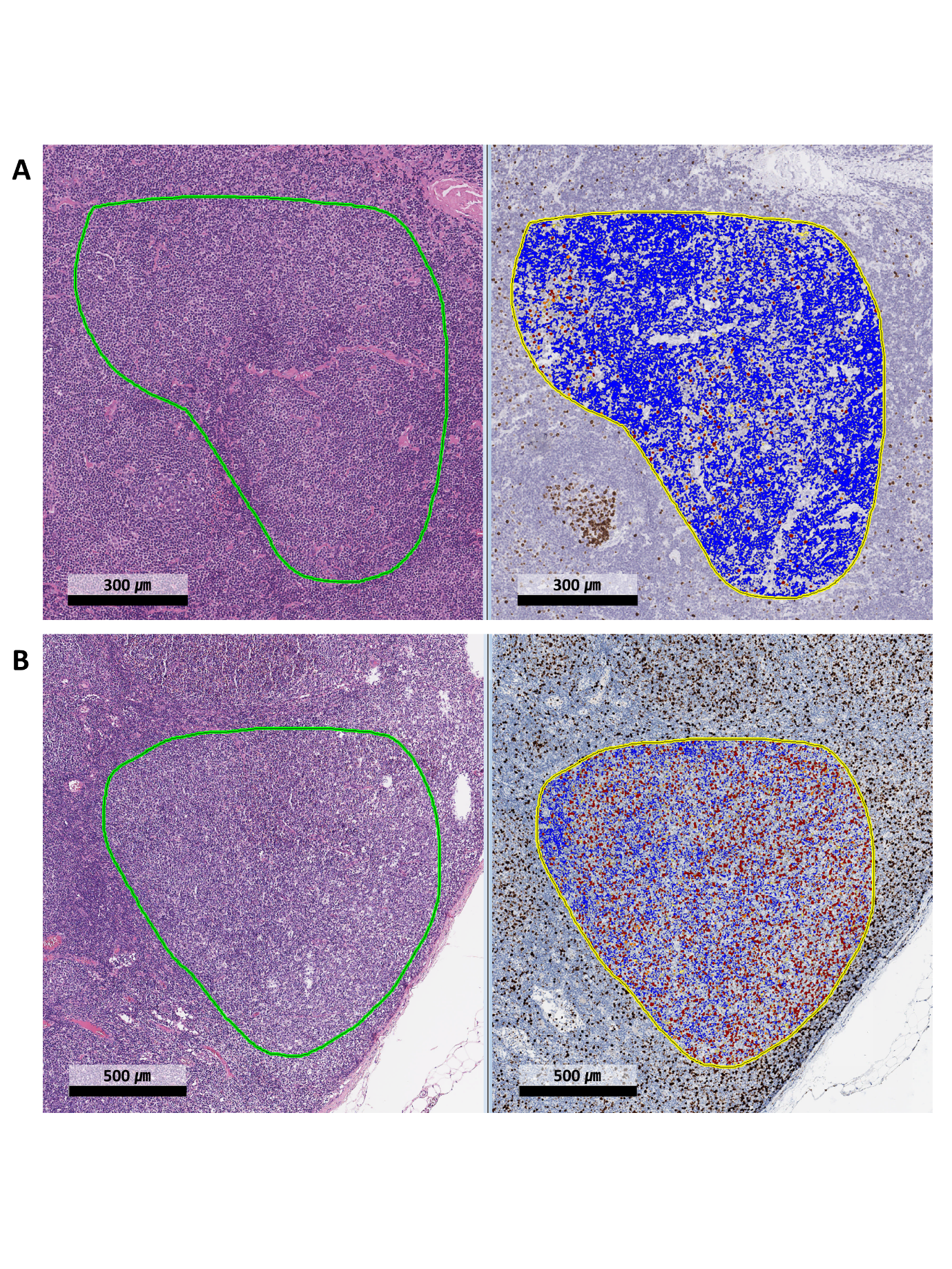


Figure S4. Quantification of the Ki-67 proliferation index by image analysis.

(A) When determining the Ki-67 proliferation index in a case (NMZL28) with residual, partially regressed germinal centers (GCs), we carefully excluded the GC areas to prevent overestimation. (B) In a case (NMZL38) with nodal architecture effacement without residual GC-like structures, image analyses were performed by selecting areas composed of diffuse proliferation of monocytoid B-cells.

Figure S5. Representative cases for estimation of the large-cell component.


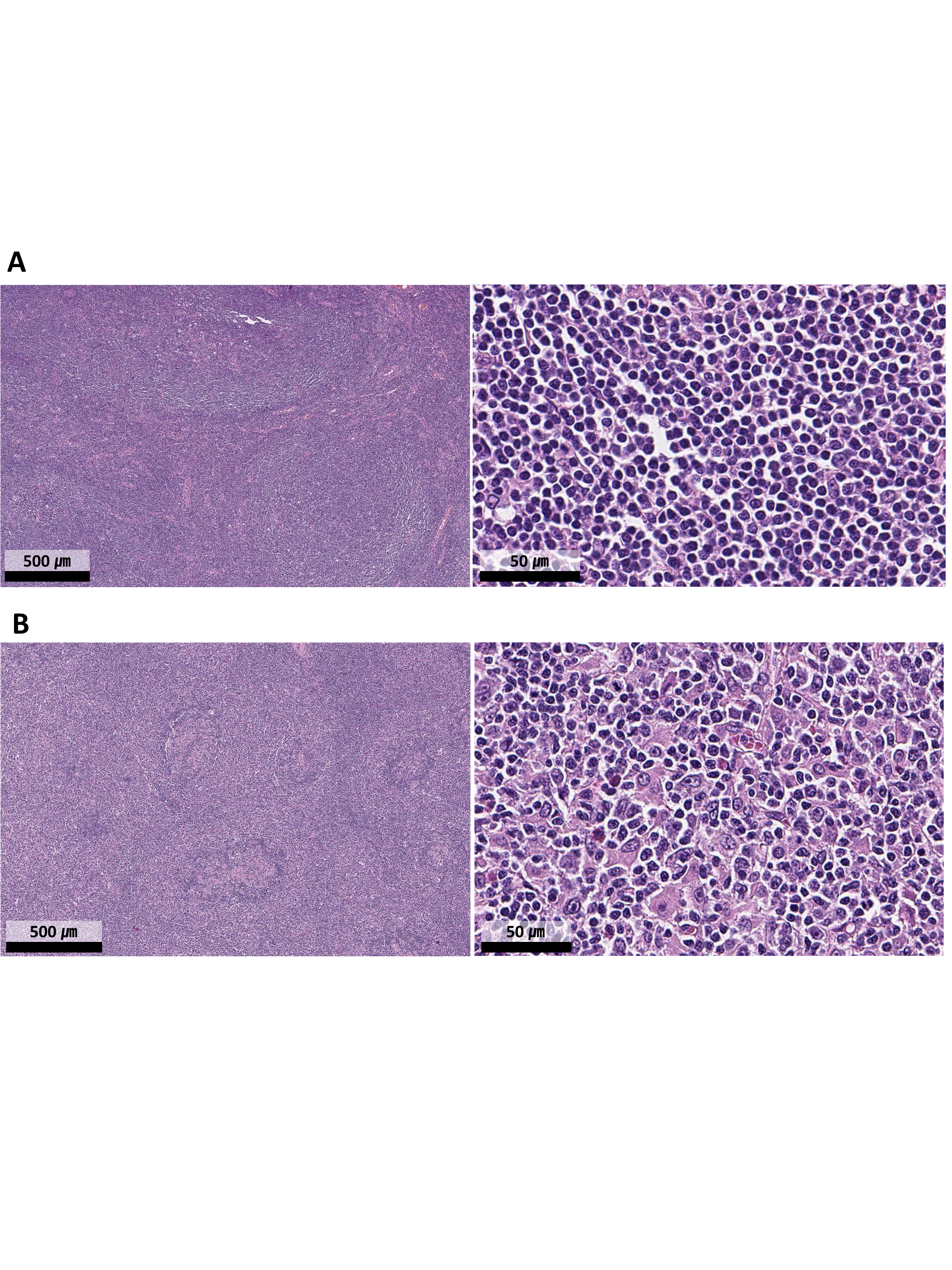


(A) NMZL22 showed a nodular structure with a prominent marginal zone, where large-cell component was estimated as 5%. (B) NMZL23 showed marked marginal zone hyperplasia, 40% of which showed abundant large monocytoid cells


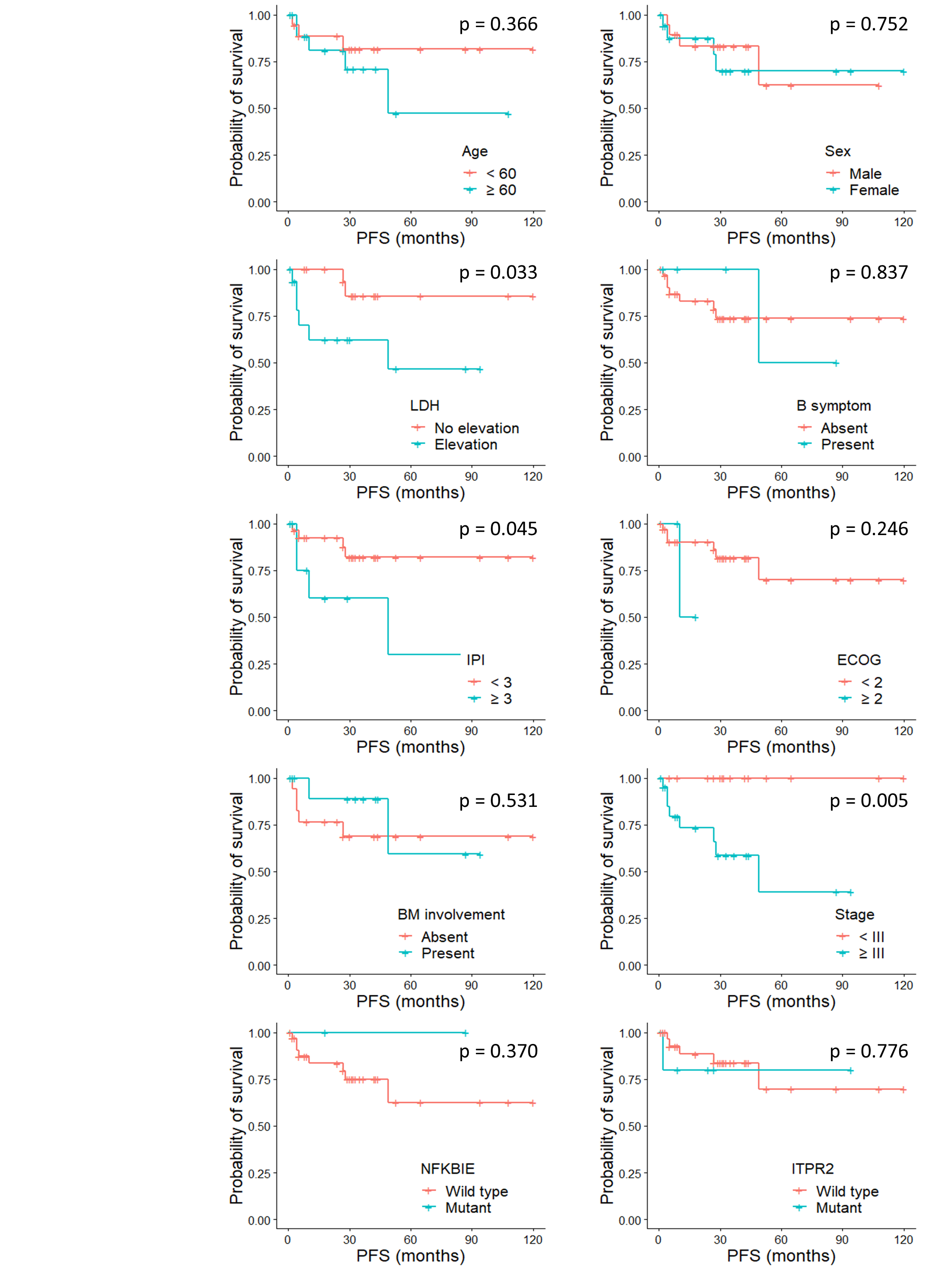


Figure S6. Progression-free survival according to various clinicopathological factors.

Figure S7. Separate survival analysis within patients treated with either R-CVP based or R-CHOP based regimen


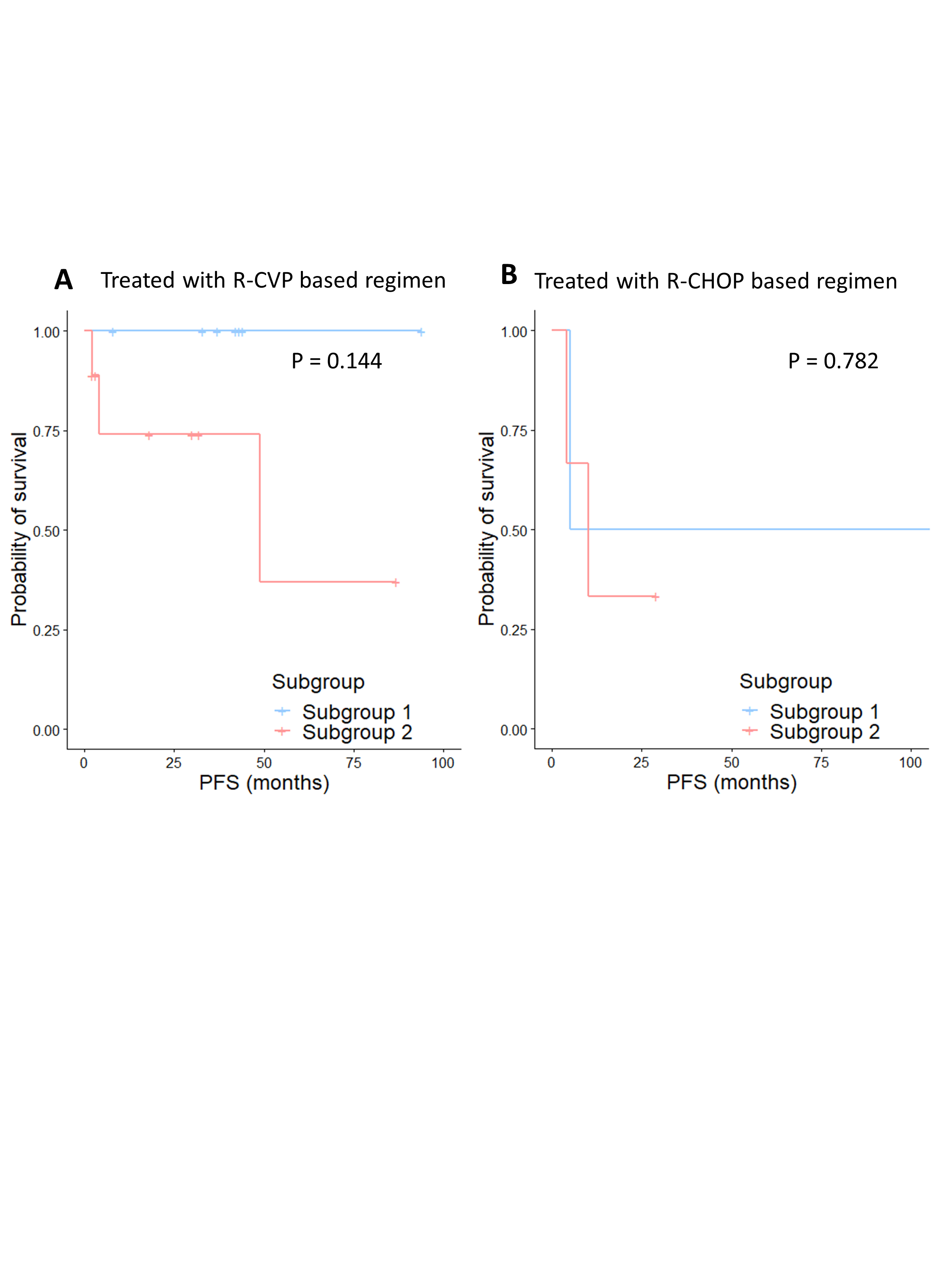

Supplement: Supplementary file 1 [file cancers-12-01669-s001.zip › SupplementaryFigures_0622.docx]
